# Sex and age differences in ICD-11 PTSD and complex PTSD: An analysis of four general population samples

**DOI:** 10.1192/j.eurpsy.2021.2239

**Published:** 2021-10-04

**Authors:** Grainne McGinty, Robert Fox, Menachem Ben-Ezra, Marylène Cloitre, Thanos Karatzias, Mark Shevlin, Philip Hyland

**Affiliations:** 1 Department of Psychology, Maynooth University, Kildare, Ireland; 2 School of Nursing, Midwifery and Health Systems, University College Dublin, Dublin, Ireland; 3 School of Social Work, Ariel University, Ariel, Israel; 4 National Center for PTSD Dissemination and Training Division, VA Palo Alto Health Care System, Palo Alto, California, USA; 5 Department of Psychiatry and Behavioural Sciences, Stanford University, Stanford, California, USA; 6 School of Health & Social Care, Edinburgh Napier University, Edinburgh, United Kingdom; 7 Rivers Centre for Traumatic Stress, NHS Lothian, Edinburgh, United Kingdom; 8 School of Psychology, Ulster University, Derry, Northern Ireland

**Keywords:** Age, complex PTSD, ICD-11, PTSD, sex

## Abstract

**Background:**

Posttraumatic stress disorder (PTSD) is traditionally understood as a disorder that occurs more commonly in women than in men, and in younger age groups than in older age groups. The objective of this study was to determine if these patterns are also observed in relation to International Classification of Diseases (ICD-11) PTSD and complex PTSD (CPTSD).

**Methods:**

Secondary data analysis was performed using data collected from three nationally representative samples from the Republic of Ireland (*N* = 1,020), the United States (*N* = 1,839), and Israel (*N* = 1,003), and one community sample from the United Kingdom (*N* = 1,051).

**Results:**

Estimated prevalence rates of ICD-11 PTSD were higher in women than in men in each sample, and at a level consistent with existing data derived from Diagnostic and Statistics Manual of Mental Disorders (DSM)-based models of PTSD. Furthermore, rates of ICD-11 PTSD were generally lower in older age groups for men and women. For CPTSD, there was inconsistent evidence of sex and age differences, and some indication of a possible interaction between these two demographic variables.

**Conclusions:**

Despite considerable revisions to PTSD in ICD-11, the same sex and age profile was observed to previous DSM-based models of PTSD. CPTSD, however, does not appear to show the same sex and age differences as PTSD. Theoretical models that seek to explain sex and age differences in trauma-related psychopathology may need to be reconsidered given the distinct effects for ICD-11 PTSD and CPTSD.

## Introduction

There are two systems used by mental healthcare professionals to diagnose trauma-related disorders: the fifth edition of the *Diagnostic and Statistics Manual of Mental Disorders* (DSM-5) [1] and the 11th version of the *International Classification of Diseases* (ICD-11) [2]. The former describes Posttraumatic Stress Disorder (PTSD) using 20 symptoms categorized into four clusters (Intrusions, Avoidance, Negative Alterations in Cognitions and Mood [NACM], and Hyperarousal), while the latter includes two related-but-distinct disorders of PTSD and complex PTSD (CPTSD). ICD-11 PTSD includes 6 symptoms distributed across three clusters (Reexperiencing in the here and now, Avoidance, and Sense of Threat) and ICD-11 CPTSD includes 12 symptoms; the 6 PTSD symptoms and 6 “Disturbance in Self-Organization” (DSO) symptoms which are distributed across three symptom clusters (Affect Dysregulation, Negative Self Concept, and Difficulties in Relationships). Thus, DSM-5 includes a broad array of trauma-specific and nonspecific symptoms under a single diagnostic category, while ICD-11 distinguishes trauma-specific and nonspecific symptoms into discrete diagnostic categories, each with a narrow set of symptom indicators. Decades of research with DSM-based models indicates that PTSD is more common among females than males, and among younger rather than older cohorts, however, it is unknown whether these sex and age differences occur with respect to ICD-11 PTSD and CPTSD.

Epidemiological research with DSM-IV [3] and DSM-5 [1] models of PTSD consistently found that women were about twice as likely as men to meet diagnostic criteria for PTSD [4–7], even when controlling for differences in trauma type, diagnostic measures, culture, measurement error, reporting bias, and file drawer effects [4,7–9]. A study with Danish bank employees exposed to several robberies found that a combination of pre, peri and posttraumatic risk factors that were more commonly reported by women accounted for 83% of the variance in the association between sex and PTSD [10]. Similarly, a recent systematic review of 19 studies found that a combination of genetic predisposition, hormonal influences, and gender roles combine to leave women at higher risk of developing PTSD [11]. There is a smaller, but nonetheless substantial body of evidence showing that rates of PTSD tend to decline in older age, with lowest rates being observed amongst those aged 65 and older [6,12–15]. Several explanations have been offered for this, including an increased risk of early mortality due to PTSD [1,6,1,7], under reporting of symptoms due to fears of stigma in older age groups [18–2,2], and greater resilience against adverse situations in older age [23–28].

With an ever-growing number of studies using the ICD-11 models of PTSD and CPTSD [29,30], it is important that to determine if traditionally understood sex and age differences in trauma-related psychopathology are observed in the context of ICD-11 PTSD and CPTSD. In this study, we reanalyzed data from four general population samples to determine if there are consistent sex and age differences in rates of ICD-11 PTSD and CPTSD.

## Methods

### Sample and procedures

This study utilized data from four existing general population, internet-based surveys from the US, the Republic of Ireland, Israel, and the UK. The US sample was collected by the survey company GfK; the Irish and UK samples were collected by the survey company Qualtrics; and the Israeli sample was collected by the survey company Ipanel. In every case, participants were recruited from existing, double opt-in research panels. Each survey lasted 20–30 min, and in every case, participants were required to be aged 18 years or older, living in their respective country, and to provide informed consent. The Israeli and US data were collected in 2017, the UK data in 2018, and the Irish data in 2019. Ethical approval for the collection of each dataset, and use for secondary analyses, was obtained by the various study authors from their respective institutions. Ethical approval for this study was provided to the first author by the Social Research Ethics Committee at Maynooth University.

The US sample (*N* = 1,839) was a nationally representative, probability-based sample of adults aged 18–70 years. In addition to the inclusion criteria previously mentioned, participants were also selected if they had experienced at least one traumatic life event. Furthermore, females and ethnic minority groups (African American and Hispanic) were oversampled, each at a 2:1 ratio. 3,953 people were contacted and 1,839 met the inclusion criteria (participation rate = 46.3%). These data were weighted to take account of all inclusion criteria and ensure representativeness to the entire US adult population. Further details can be found in Cloitre et al. [31].

The Irish (*N* = 1,020) and Israeli (*N* = 1,003) samples were nationally representative, nonprobability-based adult samples. Quota sampling methods were used to construct samples that represented the respective populations in relation to several demographic variables (i.e., age, sex, and regional distribution). All Israeli participants were trauma-exposed [32] while 82.3% of the Irish participants met the DSM-5’s Criterion A trauma exposure criterion [33]. Further details about these samples can be found in Ben-Ezra et al. [32] and Hyland et al. [33].

The UK sample (*N* = 1,051) was a community sample of trauma-exposed adults. Exposure to a traumatic life event was an inclusion criterion, and although age and regional quotas were used to select sample participants, this sample was not constructed to be nationally representative. Further details about this sample can be found in Karatzias et al. [34]. Table 1 presents the sociodemographic characteristics for each sample.

**Table 1.** Demographic characteristics of each sample.

	Ireland (*N* = 1,020)	US (*N* = 1,839)	Israel (*N* = 1,003)	UK (*N* = 1,051)
Age, Mean (SD)	43.10 (15.2)	44.51 (14.5)	40.62 (14.5)	47.13 (14.9)
Age range	18–87	18–70	18–70	18–90
Age bands (%)				
18–24	12.3	10.0	15.9	5.1
25–34	20.2	20.7	25.8	21.0
35–44	23.5	19.0	19.8	18.3
45–54	29.2	18.5	16.5	21.6
55–64	14.1	21.6	15.8	19.7
65+	10.8	10.2	6.3	14.3
Sex (%)				
Men	49	48	48.3	31.6
Women	51	52	51.7	68.4
Relationship status (%)				
Not in a committed relationship	30.5	36.6	29.5	29.6
In a committed relationship	69.5	63.4	70.5	70.4
Highest level of education (%)				
Primary school or less	7.1	9.1	2.5	1.7
High school/secondary school	39.2	28.7	29.1	35.7
College/University	36.9	30.3	68.4	62.7
Postgraduate^a^	16.9	31.8	–	–
Employment (%)				
Full-time	45.8	71.1	61.8	36.1
Part-time	17.8	–	20.9	19.2
Voluntary work^a^	–	–	–	3.2
Unemployed, seeking work	8.6	5.6	6.3	7.1
Unemployed, not seeking work	27.7	23.3	11.0	34.3

a Items regarding postgraduate qualification and voluntary work were not included in demographic questions for countries with blank spaces.

### Materials

#### Trauma exposure

The Life Events Checklist for DSM-5 [33] (LEC-5) was used to screen for traumatic exposure in the US, Israeli, and UK samples. The LEC-5 includes descriptions of 16 traumatic life events and participants were asked to indicate if they had experienced each event on a “Yes” (1) or “No” (0) basis. The International Trauma Exposure Measure (ITEM) [35] was used to screen for traumatic exposure in the Irish sample. The ITEM was developed to capture traumatic exposure in a manner that is consistent with the ICD-11’s broader definition of a traumatic event (i.e., any event of an extremely threatening or horrific nature). It includes descriptions of 21 events, 16 of which meet the DSM-5’s definition of a traumatic event and five that meet the ICD-11, but not DSM-5, definition (i.e., stalking, bullying, emotional abuse, emotional neglect, and physical neglect). To ensure consistency across all samples, in this study we only used the 16 events from the ITEM that match the DSM-5 definition of trauma. These events map onto the 16 events in the LEC-5. Thus, diagnostic rates for all samples are reported based on a traumatic exposure criterion that is in-line with the DSM-5’s Criterion A definition [36].

#### PTSD and CPTSD

All samples completed the International Trauma Questionnaire [3,7]. This 12-item, self-report measure was designed to capture all elements of the ICD-11 diagnostic criteria for PTSD and CPTSD. Respondents first identify their most distressing traumatic event and indicate how long ago it occurred. Respondents are then instructed to answer all questions in relation to this event. Six items measure PTSD symptoms, and these items are answered in terms of how much the respondent has been bothered in the past month. Three questions measure functional impairment associated with these symptoms in the domains of social, occupation, and other important areas of life. A further six items measure the DSO symptoms, and these are answered in terms of how respondents typically feels, think about themselves, and relates to others. There are three items that measure functional impairment associated with these symptoms too. All items are based on a five-point Likert scale from 0 (*Not at all*) to 4 (*Extremely*), and a symptom is considered to be present based on a score of ≥ 2 (*Moderately*). The internal reliability (Cronbach’s alpha) estimates of the subscale scores in each sample were all greater than *α* = 0.90.

To meet diagnostic criteria for PTSD or CPTSD, a person must have experienced at least one traumatic event. For a diagnosis of PTSD, at least one symptom must be present from each PTSD cluster, and at least one indicator of functional impairment associated with these symptoms must be endorsed. To meet diagnostic criteria for CPTSD, at least one symptom must be present from the six symptom clusters, and endorsement of functional impairment associated with the PTSD and DSO symptoms must be present. As per the ICD-11 diagnostic guidelines, a person may only be diagnosed with PTSD or CPTSD, but not both. If a person meets the diagnostic criteria for CPTSD, they do not also receive a diagnosis of PTSD. Diagnostic rates presented in this study represent those from the finalized version of the ITQ, and consistent with the ICD-11 diagnostic algorithms.

### Statistical analysis

Binary logistic regression analysis was used to determine if there were statistically significant differences in the estimated prevalence rates of PTSD and CPTSD across the sexes, and across six age categories (18–24, 25–34, 35–44, 45–54, 55–64, and 65 and older). These age categories were used because these were the age bands employed by the various survey companies to establish sample quotas. Odds ratios (OR) with 95% confidence intervals were estimated to quantify the magnitude of the sex and age differences. For sex, males were used as the reference category, and for age, those aged 65 years and older were used as a reference category.

## Results

The mean number of traumatic life events in the US sample was 3.77 (*Mdn* = 3.00, SD = 2.65); in the Irish sample, the mean number was 3.26 (*Mdn* = 3.00, SD = 3.18); in the Israeli sample, the mean number was 4.07 (*Mdn* = 4.00, SD = 2.77); and in the UK sample, the mean number was 3.18 (*Mdn* = 2.00, SD = 2.61).

The estimated prevalence rates of PTSD and CPTSD, and the differences between males and females, are presented in Table 2. There were statistically significant differences (*p* < 0.05) in the rates of PTSD between males and females in every sample. Females were between 1.73 (Israel) and 2.56 (US) times more likely to meet diagnostic criteria for PTSD. With respect to CPTSD, a statistically significant (*p* < 0.01) sex difference was present only in the US sample where females were 1.84 times more likely to meet diagnostic criteria.

**Table 2.** Sex differences in estimated prevalence rates of ICD-11 PTSD and CPTSD using binary logistic regression.

	PTSD % (*n*)	Males % (*n*)	Females % (*n*)	OR (95% CI)	CPTSD % (*n*)	Males % (*n*)	Females % (*n*)	OR (95% CI)
Ireland	5.0 (51)	3.6 (18)	6.3 (33)	1.82 (1.00–3.26)	7.7 (79)	6.6 (33)	8.8 (46)	1.37 (0.86–2.19)
US	3.4 (62)	1.9 (17)	4.8 (45)	2.56 (1.46–4.53)	3.9 (70)	2.7 (24)	4.9 (46)	**1.84 (1.11–3.05)**
Israel	6.7 (67)	5.0 (24)	8.3 (43)	1.73 (1.03–2.90)	4.9 (49)	5.2 (25)	4.6 (24)	0.89 (0.50–1.58)
UK	5.3 (56)	3.0 (10)	6.4 (46)	2.20 (1.10–4.41)	12.9 (136)	13.0 (43)	12.9 (93)	1.00 (0.68–1.47)

*Note:* Statistically significant (*p* < 0.05) effects are in bold.

Abbreviations: OR, odds ratios; 95% CI, 95% confidence intervals.

**Figure 1.**
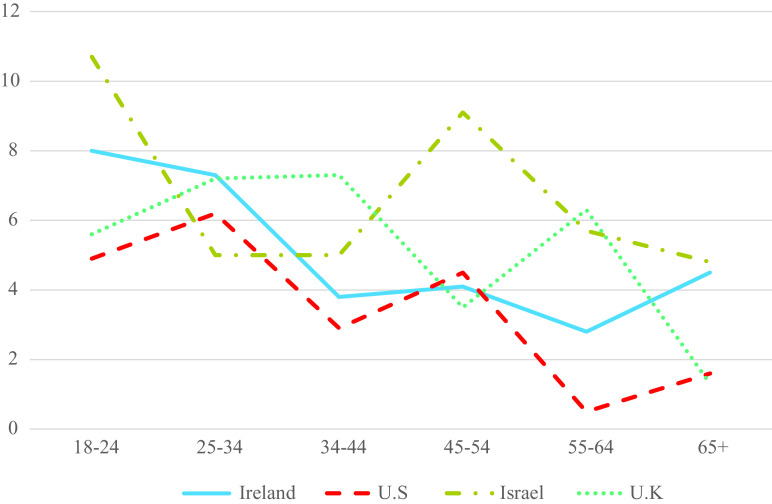
Estimated prevalence rates of ICD-11 PTSD across age groups in each sample.

**Figure 2.**
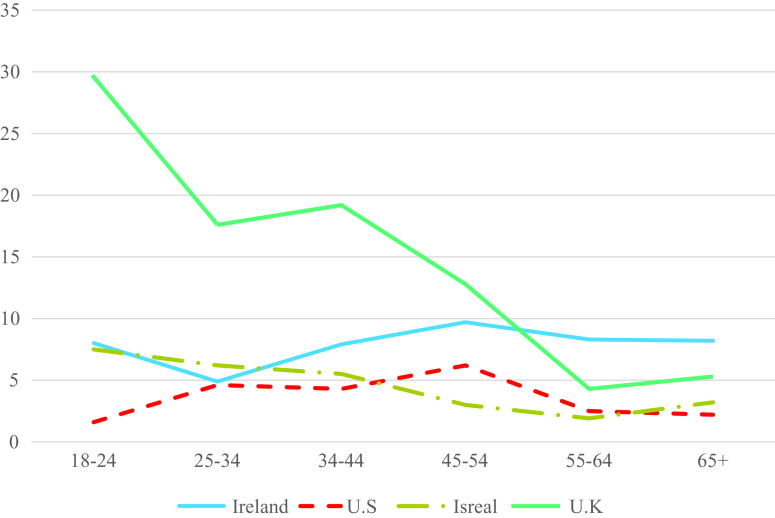
Estimated prevalence rates of ICD-11 CPTSD across age groups in each sample.

Age differences in rates of PTSD and CPTSD are presented in Table 3. There was a statistically significant age effect for PTSD (*p* < 0.001) and CPTSD (*p* < 0.05) in the US sample, and a statistically significant effect for CPTSD (*p* < 0.001) in the UK sample. In the US, individuals aged 25–35 were 4.19 times more like than those aged 65+ to meet criteria for PTSD; while those aged 44–54 were 3.32 more likely to meet criteria for CPTSD. In the UK, all age groups other than those aged 55–64 were significantly more likely than those aged 65+ to meet criteria for CPTSD with ORs ranging from 2.60 to 7.47. The rates of PTSD and CPTSD in each age group and in all samples are presented in Figures 1 and 2. As can be seen, there was a consistent trend of lower rates of PTSD in the older age groups, whereas there was little evidence of consistent differences in rates of CPTSD across the age groups.

**Table 3.** Binary logistic regression analysis predicting likelihood of receiving PTSD and CPTSD diagnosis across age groups.

	PTSD % (*n*)	*B*	SE	OR (95% CI)	CPTSD % (*n*)	*B*	SE	OR (95% CI)
Ireland								
18–25	8.0 (10)	0.60	0.56	1.82 (0.60–5.52)	8.0 (10)	−.02	0.47	0.976 (0.38–2.50)
25–35	7.3 (15)	0.50	0.53	1.65 (0.58–5.66)	4.9 (10)	−0.55	0.47	0.537 (0.23–1.45)
35–44	3.8 (9)	−0.20	0.57	0.82 (0.27–2.50)	7.9 (19)	−0.03	0.42	0.965 (0.42–2.20)
44–54	4.1 (8)	−0.10	0.53	0.90 (0.29–2.82)	9.7 (19)	0.19	0.42	1.21 (0.52–2.82)
55–65	2.8 (4)	−0.51	0.68	0.60 (0.16–2.29)	8.3 (12)	0.02	0.46	0.600 (0.15–2.29)
65+	4.5 (5)	–	–	–	8.2 (9)	–	–	–
US								
18–25	4.9 (9)	1.1	0.68	3.13 (0.82–11.92)	1.6 (3)	−0.18	0.80	0.83 (0.17–3.93)
25–35	6.2 (23)	**1.4**	**0.62**	**4.19 (1.23–14.23)**	4.6 (17)	0.92	0.59	2.50 (0.78–7.89)
35–44	2.9 (10)	0.6	0.67	1.79 (0.48–6.67)	4.3 (15)	0.85	0.59	2.33 (0.72–7.47)
44–54	4.5 (15)	1.0	0.64	2.91 (0.82–10.27)	6.2 (21)	**1.2**	**0.58**	**3.32 (1.06–10.35)**
55–65	0.5 (2)	−1.2	0.95	0.28 (0.04–1.82)	2.5 (10)	0.26	0.62	1.30 (0.38–4.40)
65+	1.6 (3)	–	–	–	2.2 (4)	–	–	–
Israel								
18–25	10.7 (17)	0.87	0.66	2.39 (0.67–8.87)	7.5 (12)	0.91	0.78	2.49 (0.54–11.45)
25–35	5.0 (13)	0.05	0.67	1.06 (0.29–3.83)	6.2 (16)	0.70	0.76	2.00 (0.45–8.97)
35–44	5.0 (10)	0.06	0.68	1.05 (0.28–3.97)	5.5 (11)	0.57	0.78	1.79 (0.38–8.28)
44–54	9.1 (15)	0.70	0.65	2.00 (0.56–7.16)	3.0 (5)	−0.05	0.85	0.953 (0.18–5.04)
55–65	5.7 (9)	0.20	0.68	1.21 (0.31–4.62)	1.9 (3)	−0.53	0.93	0.580 (0.09–3.62)
65+	4.8 (3)	–	–	–	3.2 (2)	–	–	–
UK								
18–25	5.6 (3)	1.4	0.93	4.35 (0.70–26.7)	29.6 (16)	2.0	0.47	**7.47 (2.98–18.77)**
25–35	7.2 (16)	1.7	0.76	5.77 (1.31–25.5)	17.6 (39)	1.3	0.40	**3.80 (1.72–8.40)**
35–44	7.3 (14)	1.8	0.76	5.82 (1.30–26.0)	19.3 (37)	1.4	0.41	**4.23 (1.90–9.40)**
44–54	3.5 (8)	0.99	0.79	2.70 (0.56–12.9)	12.8 (29)	0.95	0.41	**2.60 (1.15–5.85)**
55–65	6.3 (13)	1.6	0.76	4.95 (1.10–22.3)	4.3 (7)	−0.48	0.53	0.621 (0.22–1.75)
65+	1.3 (2)	–	–	–	5.3 (8)	–	–	–

*Note:* Statistically significant (*p* < 0.05) effects are in bold.

Abbreviations: *B*, unstandardized beta value; OR (95% CI), odds ratio with 95% confidence intervals; SE, standard error of beta.

**Figure 3.**
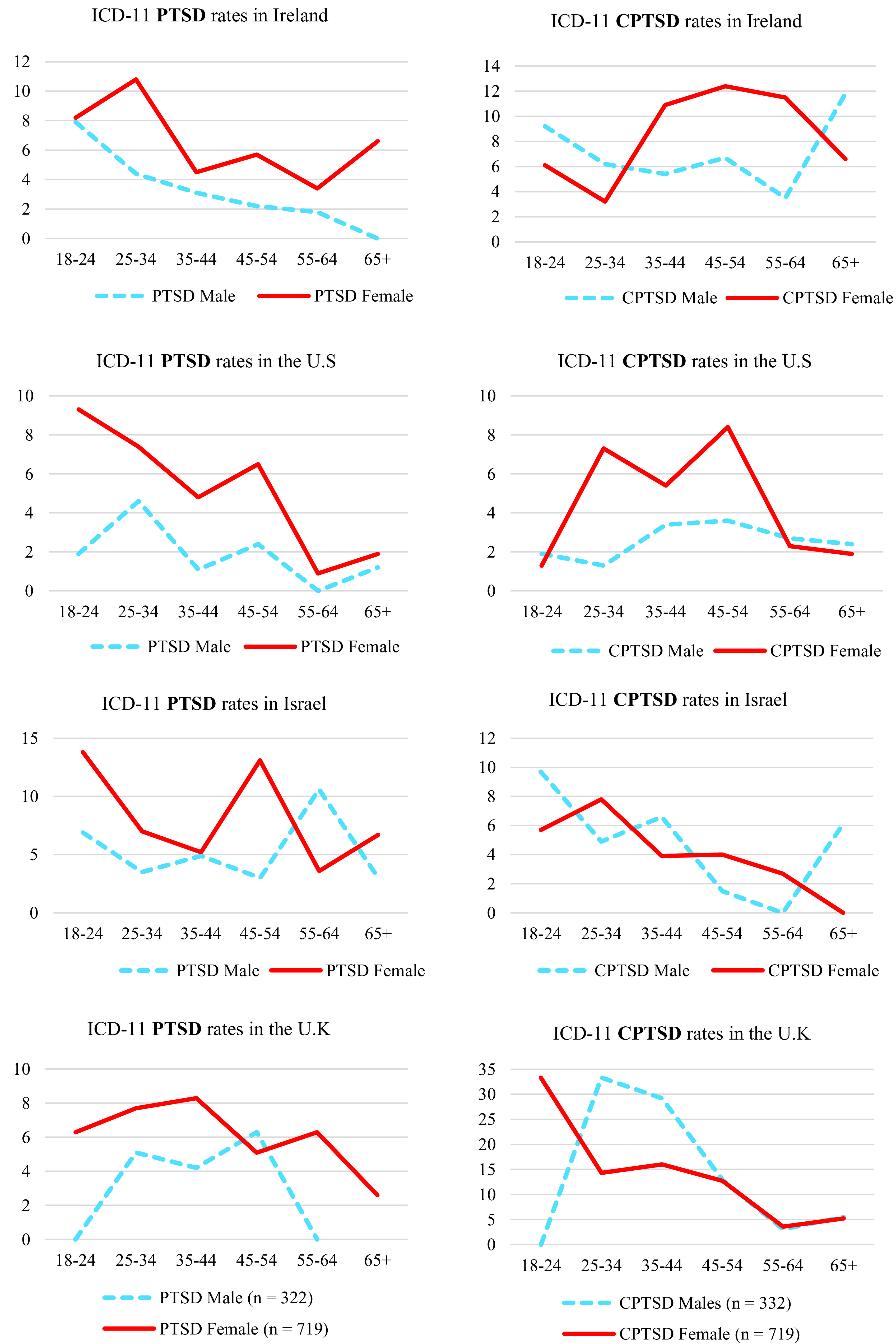
Sex differences in ICD-11 PTSD and CPTSD for Ireland, the US, Israel, and the UK illustrated across age groups.

As a post hoc exploratory analysis, the four samples were disaggregated by sex, and age differences in the rates of PTSD and CPTSD were plotted (see Figure 3). In the case of PTSD, there was a consistent trend of lower rates in the older age groups for both sexes. This was not the case for CPTSD. In the Irish and US samples, rates of CPTSD for women were highest in the middle age groups, and lowest in the youngest and oldest age groups, whereas in the Israeli and UK samples, rates were lowest for women in the oldest age groups. For males, one notable trend was that rates of CPTSD were relatively higher in those aged 65+ in the Irish and Israeli samples.

## Discussion

PTSD is normally understood as a disorder more common in women than in men, and in younger adults than in older adults. With ICD-11, PTSD was reconceptualised as a narrow, fear-based disorder, and CPTSD was introduced into the diagnostic nomenclature for the first time, sparking considerable research attention [30,38]. This necessitated a reassessment of common assumptions about sex and age differences in trauma-related psychopathology. We set out to explore these issues by reanalyzing data from four independent, general population samples. Our findings suggest that ICD-11 PTSD follows the same general sex and age profile as the DSM-based models of PTSD, while CPTSD does not.

In each sample, women had higher rates of PTSD than men, and the magnitude of these differences were consistent with previous epidemiological research using DSM models of PTSD [4–8]. We found that women were approximately two- to two-and-a-half times more likely than men to meet diagnostic criteria for ICD-11 PTSD. This is in-line with the well-established 2:1 ratio of PTSD in women compared to men [5]. So, even though PTSD is defined by a much narrower set of symptoms than in DSM-IV and DSM-5—11 and 14 fewer symptoms, respectively—the same sized sex differences in meeting diagnostic criteria appear to remain.

In contrast, in three of the four samples there were no differences in rates of CPTSD between men and women. Only in the US sample were women significantly more likely than men to meet criteria for CPTSD, with women being nearly two times more likely than men to meet diagnostic criteria. Prior to the introduction of ICD-11, sex differences in PTSD had been proposed to be due to a multitude of biopsychosocial factors including sex differences in neuroendocrine functioning following early life trauma, perceptions of threat and loss of control, peritraumatic dissociation, social isolation, and social support following traumatic experiences [5], and it had been suggested that these factors may also give rise to the same sex differences in ICD-11 CPTSD [32]. This appears not to be the case. It is unclear, however, why factors such as these would lead to sex difference in PTSD but not in CPTSD. One possibility for the observed effects could be methodological; namely that the PTSD items are biased such that women are more likely to endorse these symptoms compared to men, irrespective of their underlying levels of PTSD distress. However, this seems unlikely given that a recent comprehensive assessment found no evidence of differential item functioning based on sex for the six symptoms of ICD-11 PTSD [39]. If the current findings of sex differences in rates of PTSD but not CPTSD are evidenced in future general population surveys, a theoretical account of why such effects should occur will be required. Establishing the underlying mechanisms that contribute to higher PTSD prevalence rates in females is critical to improving treatment and prevention, globally. In doing so, this will also improve sex- and gender-specific approaches to helping those affected by trauma as well as gender-sensitive outreach, engagement, and intervention programs [1,1].

In terms of age differences in the rates of PTSD, there was only a statistically significant effect observed in the US sample, however, and as illustrated in Figure 1, there was a clear trend of lower rates of PTSD in the older age groups. This pattern is consistent with existing data from DSM-based models of PTSD, and with research on the prevalence of other psychiatric disorders across the lifespan [1,2]. Thus, we may say with some confidence that ICD-11 PTSD is like DSM-based PTSD in that prevalence rates are higher in younger age groups than in older age groups. Interestingly, quite a different pattern emerged for CPTSD. As illustrated in Figure 2, in the UK sample there was the typical profile of the highest rates in the youngest age groups and the lowest rates in older age groups. There was also some evidence of this in the Israeli sample (e.g., a drop from 7.5% in those aged 18–14 to 3.2% in those aged 65 and older). However, in the Irish sample, rates of CPTSD were lowest in those aged 25–34 and highest in those aged 44–54. In the US sample, rates of CPTSD were lowest amongst those aged 18–24 and highest in those aged 44–54 years.

Many theories have been proposed to account for decreasing rates of PTSD in older age. Compared to younger adults, when faced with adverse situations and stressful events older adults are generally more resilient and have greater cognitive reappraisal capacities [23–25,28]. Theories such as the socioemotional selectivity theory suggest that older adults seek emotionally meaningful goals and select familiar social partners which decreases the likelihood of experiencing stressful situations and increases positive experiences [1,2]. It has also been suggested that old age is associated with spending more time in quiet reflection, a decreased interest in superfluous social interactions, and acceptance of earlier life events [40]. Furthermore, there have been contrasting arguments asserting that older adults may be more reluctant to acknowledge mental health concerns due to fears of stigma and to convey their psychological concerns as somatic complaints [18–22]; to under report symptoms due to cognitive impairment [2,8]; and to possibly reflect a survivor bias where older adults are far less likely to survive until old age with a PTSD diagnosis [19,41]. Additionally, there have been concerns regarding the accuracy of psychiatric assessments in older adults given that older adults may not fit easily into our existing disorder classification systems [42]. As with the discussion of sex differences, why these processes would lead to lower rates of PTSD in older age but not in CPTSD is unclear. One possibility is that because CPTSD is more likely to occur following early developmental trauma and/or multiple traumas [33,34,43] and is associated with greater comorbidity and difficulties in functioning than PTSD [34,44] it may remit as commonly as PTSD in older age.

When age differences in rates of PTSD and CPTSD were examined separately for men and women, we found that the general pattern of lower rates of PTSD in older age groups was present for men and women. Thus, there appears to be little evidence of any interaction between sex and age in relation to PTSD. As such, it may be said with reasonable confidence that women are at higher risk of PTSD than men irrespective of age, and that rates of PTSD are generally higher in younger age groups irrespective of sex. In the case of CPTSD, however, there were signs of an interaction between sex and age. For example, in Ireland, rates of CPTSD in women followed an n-shaped distribution peaking in the middle-aged groups whereas for men, rates of CPTSD were elevated in those 18–24, were lower in all age groups up to those aged 55–64, and then were at their highest in those aged 65 and older. A similar pattern was evident in the US sample save for the high rates of CPTSD in men over 65; an effect that may due to the fact that the US sample only included adults up to the age of 70. In Israeli, rates of CPTSD for men and women were similar for every age group before a stark difference becoming evident in those aged 65 and older where men had considerably higher rates. Almost the opposite pattern was evident in the UK where rates of CPTSD were starkly different between men and women aged 18–24 and were then very similar among the middle- older-aged groups. Consequently, these findings suggest that rates of CPTSD at different ages may depend on one’s sex.

These findings should be considered in light of some limitations. The four samples were drawn from high-income countries and findings may not generalize to other nations. The use of general population samples means that these findings may not generalize to clinical populations. Relatedly, only the US sample was a probability-based nationally representative sample. The sample sizes were relatively small when attempting to categorize people into different age groups, and this likely increased the risk of Type 2 errors when testing for age differences. Finally, the cross-sectional nature of the sample means that it is impossible to disentangle age versus cohort effects. We may only conclude that rates differ across age groups but not those rates change because of the aging process.

In conclusion, we found consistent evidence that rates of ICD-11 PTSD were higher in women than in men, and at a level that was consistent with existing data derived from DSM-based PTSD research. Moreover, also in-line with DSM-based research, rates of ICD-11 PTSD followed a general trend of decreasing frequency in older age for both men and women. The picture for CPTSD was quite distinct with inconsistent evidence of sex and age differences, and some indication of an interaction between these two demographic variables. More research is required to understand the epidemiology of CPTSD, and theoretical models of sex and age differences in trauma-related psychopathology may need to be reconsidered in light of distinct effects for PTSD and CPTSD.

## Data Availability

The data that support the findings of this study are available from the corresponding author upon reasonable request.
